# Using Dietary Macronutrient Patterns to Predict Sarcopenic Obesity in Older Adults: A Representative Korean Nationwide Population-Based Study

**DOI:** 10.3390/nu13114031

**Published:** 2021-11-11

**Authors:** Jun-Hyuk Lee, Hye-Min Park, Yong-Jae Lee

**Affiliations:** 1 Department of Family Medicine, Nowon Eulji Medical Center, Eulji University School of Medicine, Seoul 01830, Korea; swpapa@eulji.ac.kr; 2 Department of Family Medicine, Chaum Medical Checkup Center Samseongdong Branch, Cha University, Seoul 06125, Korea; m213001@chamc.co.kr; 3 Department of Family Medicine, Yonsei University College of Medicine, Gangnam Severance Hospital, Seoul 06273, Korea

**Keywords:** energy intake, dietary carbohydrates, muscle, skeletal, obesity

## Abstract

Older adults with sarcopenic obesity (SO) are at increased risk of adverse health outcomes. It has not been identified which pattern of macronutrient intake is appropriate in relation to SO. We aimed to compare the patterns of macronutrient intake for predicting SO in older adults. Data from a total of 3828 older adults who participated in the 2008–2011 Korea National Health and Nutrition Examination Survey were analyzed. The one-day 24 h dietary recall method was used to assess macronutrient intake. SO was defined by a combination of body mass index (BMI) ≥ 25 kg/m^2^ and BMI adjusted-appendicular skeletal muscle mass <0.789 for men and <0.512 for women. Weighted logistic regression analysis revealed the odds ratio (95% confidence interval) for SO of total calorie intake per 100 increments and carbohydrate (CHO) intake (g/kg/day) per 1 increment to be 0.95 (0.91–0.99) and 0.83 (0.74–0.94), respectively, after adjusting for confounding variables in women. The predictive power for SO of CHO intake (g/kg/day) was higher compared with the other patterns of macronutrient intake both in men and women. In conclusion, total calorie intake and CHO intake (g/kg/day) are inversely related to SO in women. CHO intake (g/kg/day) could be the best index for determining SO.

## 1. Introduction

The global population is progressively aging. Approximately 22% of the entire population will be over 60 years of age in 2050, while approximately 5% will be over 80 years of age [1]. South Korea is one of the most rapidly aging countries in the world. At the end of 2019, the proportion of people aged 65 and over was 15.5 percent of the total population. According to Statistics Korea, South Korea will become the world’s oldest society by 2067, with older adults accounting for 46.5 percent of the population [2].

Changes in body composition with aging are characterized by a decrease in muscle mass and a concomitant increase in fat mass, with or without changes in body weight [3,4,5]. With the increasing numbers of older adults, health-related problems resulting from body composition changes are also increasing. A loss of muscle mass, muscle strength, and/or reduced physical performance, called sarcopenia, is related to the risk of frailty and its related complications, such as falls and fractures [6,7,8]. Moreover, an increase in fat mass, the central redistribution of fat, and obesity contribute to the risk of metabolic disorders such as hypertension, type 2 diabetes mellitus, and cardiovascular disease [9,10,11]. Older adults with sarcopenic obesity (SO) are at especially high risk of those adverse outcomes compared to either those with sarcopenia or obesity alone due to the synergistic effects of sarcopenia and obesity in a proinflammatory state [6,12]. In addition, recent evidence implies that SO is independently associated with cognitive impairment and the non-remission of late-life depression [13,14]. Therefore, prevention of SO is critical for public health and the maintenance of physical wellness, mental health, and quality of life.

Multiple factors contribute to changes in body composition, including age, ethnicity, physical activity, socioeconomic status, smoking, alcohol intake, pharmacological agents (e.g., prednisone, insulin, sulfonylureas, tricyclic anti-depressants), and dietary intake [15]. Dietary proteins in particular offer essential amino acids for muscle protein synthesis while also functioning as an anabolic stimulus, with specific effects on protein synthesis [16]. Meanwhile, interventions of caloric restriction, high intakes of dietary fiber, and proper meal timing are effective strategies for obesity management [17,18,19,20]. It remains controversial, however, whether eating a high-carbohydrate (CHO) diet or consuming a higher percentage of total energy from CHO composition increases the risk of obesity [21]. According to the Korean Geriatric Society and the Korean Nutrition Society’s protein intake recommendation for older Korean adults to prevent sarcopenia [22], a daily protein consumption of >1.2 g per body weight is recommended in older adults, with the exception of those with substantial kidney impairments. Only a few studies to date have suggested that macro-nutritional strategies, such as enhanced protein intake, could improve physical function and weight reduction in frail adults with obesity [23,24,25]. In accordance with the 2020 Korean Society for the Study of Obesity Guidelines, even if the need for individualized calorie restriction and composition of macronutrients are mentioned in the management of obesity, it still cannot be determined which detailed patterns of macronutrient intake are recommended for the prevention of SO [26]. Although there are findings on nutritional intake that consider either sarcopenia or obesity alone, it has not been verified which pattern of macronutrient intake is appropriate when considering both muscle and fat metabolism.

Based on these perspectives, the determination of the macronutrient intake patterns most associated with SO could be an effective strategy for the prevention of SO. Therefore, this study aimed to compare the patterns of macronutrient intake for the prediction of SO in older adults using cross-sectional Korean representative nationwide population-based data.

## 2. Materials and Methods

### 2.1. Study Population

The Korea National Health and Nutrition Examination Survey (KNHANES) is a nationwide, representative, and population-based survey annually conducted by the Korea Centers for Disease Control and Prevention to monitor the health and nutritional status of the Korean population [27]. The participants in the KNHANES were sampled based on a multi-stage, stratified, cluster probability sampling, which uses the primary sampling units as defined by the geographical areas in Korea [28]. The sampling weights were assigned to each participant to generalize the units for representing the Korean population [28].

**Figure 1:**
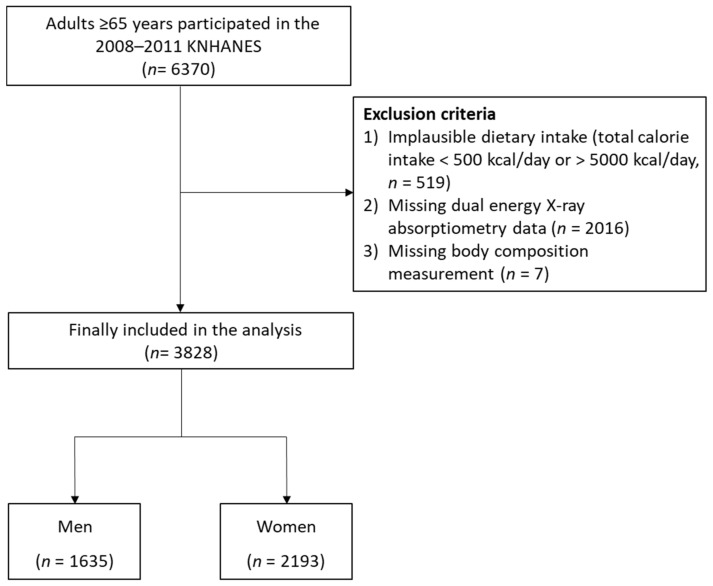
Flowchart of the study population selection.

Figure 1 presents a flowchart of the study population selection. To a total of 6370 participants ≥65 years in the 2008–2011 KNHANES, we applied the following exclusion criteria: (1) implausible dietary intake information, defined as total calorie intake <500 kcal/day or >5000 kcal/day [29] (*n* = 519); (2) missing dual energy X-ray absorptiometry (DXA) data (*n* = 2016); and (3) missing body composition measurement data (*n* = 7). Finally, the data from 3828 participants (1635 men and 2193 women) were analyzed in this study.

### 2.2. Patterns of Macronutrient Intake

The information on dietary intake was obtained using the one-day 24 h dietary recall method [30]. Daily total calorie intake (kcal/day), protein intake (g/day), CHO intake (g/day), and fat intake (g/day) were calculated. The daily total calorie intake was calculated by the summating total consumption of protein, CHO, and fat (1 g protein = 4 kcal, 1 g CHO = 4 kcal, and 1 g fat = 9 kcal) [31]. The percent of the energy intake from protein or CHO was calculated as follows: 4 kcal × protein intake (g/day)/total energy intake (kcal/day) × 100%; 4 kcal × CHO intake (g/day)/total energy intake (kcal/day) × 100%. The percentage of energy intake from fat was calculated as follows: 9 × kcal fat intake (g/day)/total energy intake (kcal/day) × 100%. Protein intake per body weight (g/kg/day), CHO intake per body weight (g/kg/day), and fat intake per body weight (g/kg/day) were also calculated.

### 2.3. Assessment of SO

Height (m) and weight (kg) were measured to the nearest 0.001 m and 0.1 kg. The body mass index (BMI) was calculated by dividing weight by the square of height (k/m^2^). According to the definition of the Korean Society for the Study of Obesity [32], a BMI ≥ 25 kg/m^2^ was considered obese. Using whole-body DXA (QDR 4500A; Hologic Inc., Bedford, MA, USA), body composition data were collected from the head, trunk, pelvic region, arms, legs, and entire body. The lean body mass (g), fat mass (g), bone mineral content (g), bone mineral density (g/cm^2^), and total fat percentage (fat mass/total mass × 100) were recorded for each anatomical region. We calculated skeletal muscle mass as lean body mass (g)—bone mineral content (g). The appendicular skeletal muscle mass (ASM) was calculated through the summation of skeletal muscle mass from both arms and legs. The skeletal muscle mass index (SMI) was defined as ASM/BMI. According to the Foundation for the National Institutes of Health (FNIH) Sarcopenia Project criteria [33], a low skeletal muscle mass index (LSMI) is defined as an SMI of less than 0.789 for men and 0.512 for women. Finally, SO was defined as concomitant LSMI and obesity.

### 2.4. Covariates

Waist circumference (cm) was measured in the horizontal plane midway between the iliac crest and lowest rib. Abdominal obesity was defined as waist circumference ≥90 cm in men and ≥85 cm in women according to the Korean-specific cut-offs for abdominal obesity [34]. Blood pressure was manually measured by well-trained nurses using mercury sphygmomanometers (Baumanometer; W.A. Baum, Copiague, NY, USA). Systolic blood pressure (SBP) and diastolic blood pressure (DBP) were defined as the average of the last two of the three measured values. The mean blood pressure (MBP) was calculated as follows: MBP = (SBP + 2 × DBP)/3. A never smoker was defined as an adult who has never smoked, or who has smoked less than 100 cigarettes in their lifetime. Former smoker was defined as an adult who has smoked at least 100 cigarettes in their lifetime and who had quit smoking at the time of interview. A current smoker was defined as any participant has smoked at least 100 cigarettes during their lifetime and who currently smokes. Current smoker was further divided into every day smoker and someday smoker whether they smoke every day or not. The level of alcohol intake (g/day) was calculated by multiplying the number of standard drinks consumed per drinking day, the amount of pure alcohol in a standard drink, and the average drinking frequency per day. The level of physical activity was assessed based on the International Physical Activity Questionnaire [35]. Regular exercise was defined as exercising vigorously ≥20 min at least 3 days/week or ≥30 min of moderate exercise/walking at least 5 days/week. Based on the Charlson comorbidity index [36], the following seven comorbid conditions were included in the list of chronic diseases: (1) stroke, (2) myocardial infarction, (3) diabetes mellitus, (4) chronic obstructive pulmonary disease, (5) chronic kidney disease stage 3 to 5, (6) liver cirrhosis, and (7) any cancers. The number of chronic diseases for each participant was classified into 3 groups: zero, one, or more than two chronic diseases. Blood samples of each participant were collected from antecubital vein after at least 8 h of fasting. Fasting plasma glucose (FPG) and serum total cholesterol concentrations were measured using a Hitachi 7600 Analyzer.

### 2.5. Statistical Analysis

We applied sampling weights to the participants to examine the Korean population’s representative data. The weights were adjusted with the values for the inverse of the response rates and the inverse of the selection probability to the sex- and age-specific values for the Korean population (post-stratification) [27]. All the data in this study are presented as mean ± standard error (SE) for the continuous variables or as percentages (SE) for the categorical variables. A weighted generalized linear regression analysis was used to compare the differences of continuous variables between participants with SO and those without SO. A weighted chi-square test was used to compare the differences of categorical variables. Restricted cubic spline curves were produced to show the dose-response relationship between macronutrient intake and SO. Weighted logistic regression analyses were performed to calculate odds ratio (OR) with a 95% confidence interval (CI) for SO of total calorie intake per 100 increments and each macronutrient intake pattern per 1 increment. Receiver-operating characteristics (ROC) were drawn and the areas under the ROC curves (AUC) were used to compare the predictive power for SO of total calories, the percentage of energy intake from macronutrients, and the macronutrient intake per body weight. All the statistical analyses were conducted using SPSS statistical software (version 25.0; SPSS Inc., Chicago, IL, USA) and R (version 4.0.3; R Foundation for Statistical Computing, Vienna, Austria). The significance level was set at *p* < 0.05.

## 3. Results

### 3.1. Clinical Characteristics of the Study Population

Table 1 presents the clinical characteristics of the older adults with or without SO. In both men and women, the mean values of BMI, waist circumference, and FPG, as well as the proportion of participants with abdominal obesity, the number of participants with one chronic disease, and the number of participants with at least two chronic diseases were higher in the participants with SO. The mean values of CHO intake per body weight, protein intake per body weight, and SMI in participants with SO was lower than that in those without SO. In men, the mean value of MBP was higher in the participants with SO compared to those without SO. In women, the mean values of the serum total cholesterol level and the percentage of fat intake were higher in the participants with SO compared to those without SO. The mean values of total calorie intake and percentage of CHO intake, as well as the proportion of participants who exercised regularly, were lower in the participants with SO than in those without SO.

**Figure 2:**
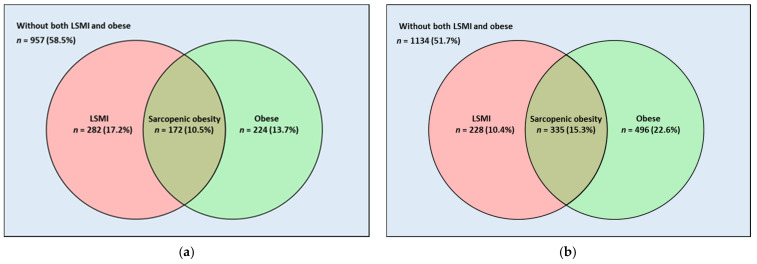
Venn Diagram showing the number of participants with LSMI, obesity, and sarcopenic obesity in (**a**) men and (**b**) women. LSMI was defined as appendicular skeletal muscle mass divided by body mass index <0.789 for men and <0.512 for women. Body mass index ≥25 kg/m^2^ was considered obese. Sarcopenic obesity was defined as concomitant LSMI and obesity. Abbreviation: LSMI, low skeletal muscle mass index.

Figure 2 shows the number and percentage of participants with LSMI, obesity, and SO status in men (Figure 2a) and women (Figure 2b) using a Venn diagram. The number and percentage of participants with LSMI, obesity, and SO were 282 (17.2%), 224 (13.7%), and 172 (10.5%) in men, and 228 (10.4%), 496 (22.6%), and 335 (15.3%) in women, respectively.

### 3.2. Relationship between Macronutrient Intake and SO

**Figure 3:**
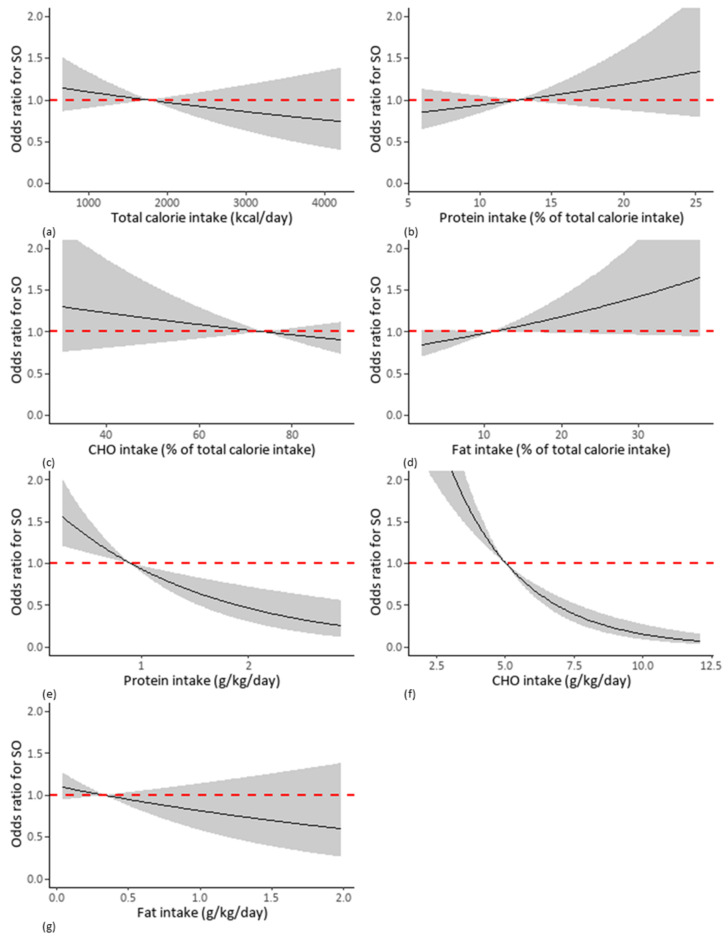
Cubic spline curves showing relationship between OR for SO and each pattern of macronutrient intake in men. Each pattern of macronutrients intake consists of (**a**) total calorie intake (kcal/day), (**b**) percentage of calorie intake from protein intake, (**c**) percentage of calorie intake from CHO, (**d**) percentage of calorie intake from fat, (**e**) protein intake per body weight, (**f**) CHO intake per body weight, (**g**) fat intake per body weight. Abbreviations: OR, odds ratio; SO, sarcopenic obesity; CHO, carbohydrate.

**Figure 4:**
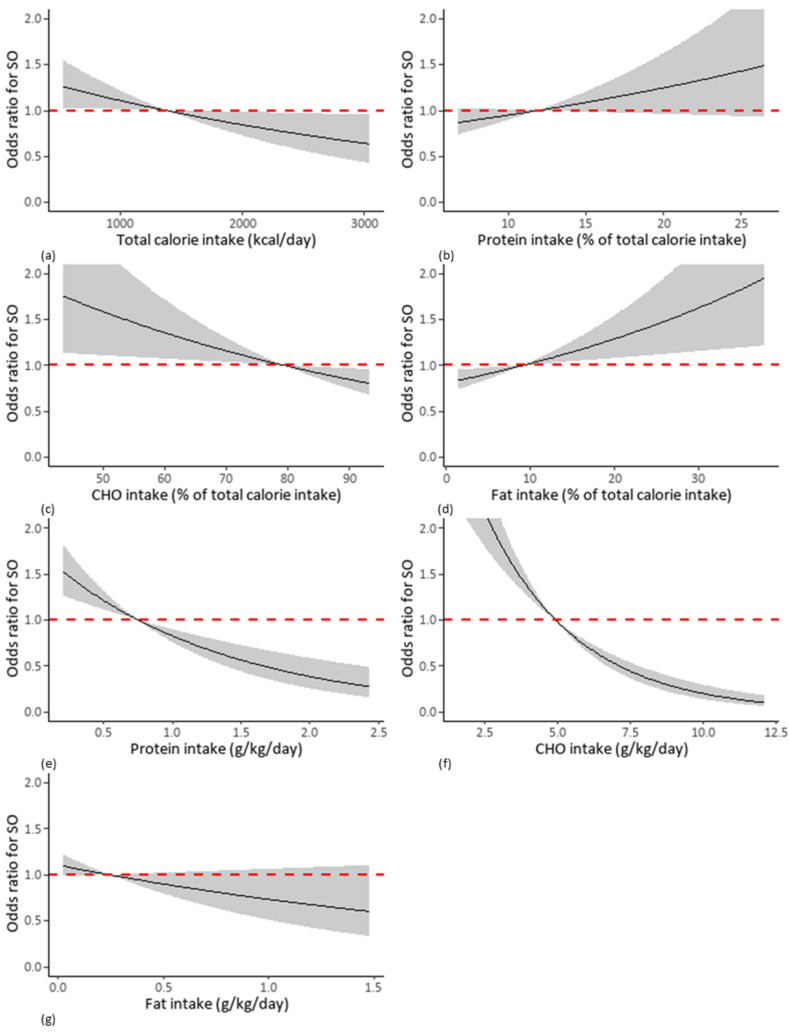
Cubic spline curves showing relationship between OR for SO and each pattern of macronutrient intake in women. Each pattern of macronutrients intake consists of (**a**) total calorie intake (kcal/day), (**b**) percentage of calorie intake from protein intake, (**c**) percentage of calorie intake from CHO, (**d**) percentage of calorie intake from fat, (**e**) protein intake per body weight, (**f**) CHO intake per body weight, (**g**) fat intake per body weight. Abbreviations: OR, odds ratio; SO, sarcopenic obesity; CHO, carbohydrate.

Figure 3 and Figure 4 present restricted cubic spline curves showing the relationship between the pattern of macronutrient intake and OR for SO in men (Figure 3a–g) and women (Figure 4a–g). There were negative dose-response relationships between the total calorie intake/percentage of CHO intake/protein intake per body weight/CHO intake per body weight/fat intake per body weight and SO, whereas the percentage of protein intake and the percentage of fat intake exhibited positive dose-response relationships with SO in both men and women.

Table 2 presents the weighted logistic regression analysis showing the relationship between patterns of macronutrient intake and SO. In men, the OR (95% CI) for SO of the percentage of fat intake per 1 increment, the protein intake per body weight per 1 increment, and the CHO intake per 1 increment were 1.02 (1.00–1.05), 0.51 (0.31–0.81), and 0.68 (0.60–0.78), respectively. After adjusting for age, waist circumference, regular exercise, smoking status, amount of alcohol intake, MBP, FPG, serum total cholesterol level, and the number of chronic diseases, there was no significant relationship between the patterns of macronutrient intake and SO. In women, the OR (95% CI) for SO of total calorie intake per 100 increments, the percentage of protein intake per 1 increment, the percentage of CHO intake per 1 increment, the percentage of fat intake per 1 increment, the protein intake per body weight per 1 increment, and the CHO intake per 1 increment were 0.96 (0.93–0.99), 1.04 (1.00–1.08), 0.98 (0.67–0.99), 1.03 (1.01–1.05), 0.43 (0.64–0.70), and 0.70 (0.64–0.77), respectively. After adjusting for the same confounding variables above, the adjusted OR (95% CI) for SO of the total calorie intake per 100 increments and the CHO intake per body weight per 1 increment were 0.95 (0.91–0.99) and 0.83 (0.74–0.94), respectively.

### 3.3. Comparison of the Predictive Power for SO of Macronutrient Intake

**Figure 5:**
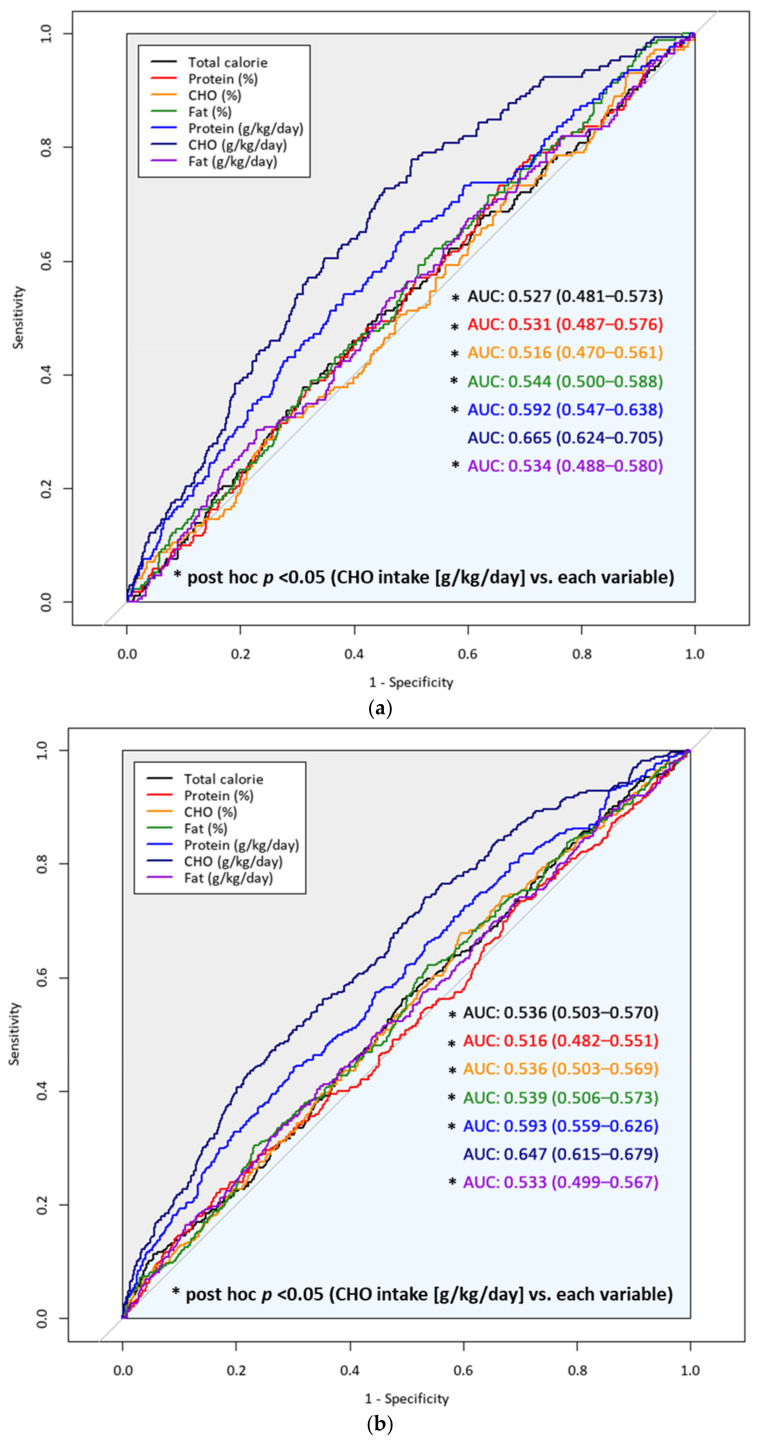
Comparison of predictive power for sarcopenic obesity of each pattern of macronutrient intake in (**a**) men and (**b**) women. Abbreviations: CHO, carbohydrate; AUC, area under the receiver-operating curve.

Figure 5 presents the comparison of the predictive power for SO of each pattern of macronutrient intake using the ROC curve graph. The AUC with 95% CI of CHO intake per body weight was 0.665 (0.624–0.705) in men and 0.647 (0.615–0.679) in women, which was higher compared with the other patterns of macronutrient intake with statistical significance in both men and women. The cut-point of CHO per body weight for SO was 5.170 in men and 3.776 in women.

## 4. Discussion

Macronutrient intake is essential for both muscle and fat metabolism. Despite the importance of macronutrient intake, there has been insufficient evidence defining the role of macronutrient intake in both muscle and fat metabolism concomitantly. Furthermore, due to age-related muscle loss and fat accumulation, determining a proper macronutrient eating pattern for preventing SO is especially critical for older adults. Therefore, we analyzed the association between SO and specific macronutrient intake patterns in older adults. The relationship between the patterns of macronutrients and SO was only significant in total calorie intake and CHO intake per body weight after adjusting for confounding variables in women. In addition, the predictive power for SO was highest in CHO intake per body weight in both men and women. The cut-off point of CHO per body weight for predicting SO was 5.170 in men and 3.776 in women, respectively. The Institute of Medicine recommends the consumption of 5–12 g of CHO per body weight depending on the physical activity level [37]. The lower limit is similar to or slightly higher than the cut-off points in this study. Considering that older adults typically have low physical activity levels, our results support the recommendation of the Institute of Medicine. In addition, since the biological differences between men and women influencing SO cannot be overlooked, all the analyses were performed separately for men and women. Most metabolic components, including glucose, fat, glucose, and energy metabolism, are sexually dimorphic. More than 3000 genes have been found to be expressed differently in male and female skeletal muscles [38]. Horton et al. demonstrated that women have higher rates of lipolysis in adipose tissue than males, probably because they are more reliant on free fatty acids as an energy source under high energy demands, such as exercise [39]. However, the molecular mechanisms through which sex hormones influence human metabolism, and thus the size of specific fat depots, are poorly understood [40].

CHO intake per body weight is the most widely utilized concept in determining the relationship between macronutrients and SO even though protein intake per body weight or percentage of CHO intake is also widely used. In light of the results of this study, however, CHO intake per body weight seems to be a parameter worth investigating in the future for its association with SO. In Table S1, after adjusting for confounding variables, total calorie intake only exhibited a significant relationship with LSMI in both men and women. On the other hand, CHO intake per body weight demonstrated a significant negative association with obesity in both men and women. These findings imply that the impact of total calorie intake on LSMI outweighs obesity, whereas CHO intake per body weight has a greater impact on obesity than LSMI. The fact that women typically have 10% higher body fat compared to men and a higher predictive power for SO of CHO per body weight compared to total calorie intake was observed in this study might explain why the relationship between CHO intake per body weight and SO was only significant in women [40].

Along with CHO intake per body weight, other accompanying nutrient intake patterns may also have influenced the outcome through muscle and fat metabolism. Table S2 demonstrates that CHO intake per body weight has a positive correlation with total calorie intake, percentage of CHO intake, and both protein and fat intake per body weight, whereas a negative correlation was observed with the percentage of protein and fat intake. In addition, the correlation coefficient was highest in total calorie intake, with an especially strong correlation in women. While total calorie intake has a positive relationship with obesity, a low total calorie intake may increase the risk of sarcopenia [41]. However, since this study confirmed that the highest predictor of SO is CHO intake per body weight through ROC comparison, it is expected that the metabolic effect of CHO intake on muscle and fat may be greater than that of total calorie intake.

Several limitations should be taken into account when interpreting the outcomes of the current study. First, the predictive power of macronutrient intake for SO was not excellent. However, it is meaningful that CHO intake per body weight demonstrated a higher predictive power for SO compared to protein intake per body weight. Moreover, we also found that CHO intake per body weight was the only variable that demonstrated a significant relationship to SO. Second, the one-day 24 h dietary recall method could be less reliable than the two-day or three-day 24 h dietary recall method. According to the results of a previous study [42], the total energy intake from one-day 24 h dietary recall in the KNHANES was under-reported, although the one-day 24 h dietary recall method was validated in older Korean women in another study [43]. Follow-up studies using the two-day or three-day 24 h dietary recall method or a food frequency questionnaire are necessary. Third, because we used secondary data from KNHANES, we could not evaluate the levels of obesity-related hormones, such as insulin, estrogens, androgens, growth hormone, and leptin, which regulate adipose tissue distribution, metabolism, and appetite. Some important data were also not available regarding the exact source and quality of the macronutrients, and meal timing. Compared with high glycemic index diets, low glycemic index diets exert a moderate effect on lowering body weight and serum C-reactive protein levels [44,45]. Animal-based proteins, meanwhile, could be beneficial for lean muscle mass compared to plant-based proteins [46]. Therefore, subsequent studies considering the source of nutrients should be conducted. Fourth, we could not define sarcopenia due to a lack of information about muscle strength and physical performance in the 2008–2011 KNHANES. Finally, a causal relationship between patterns of macronutrient intake and SO could not be verified. However, our study could provide formative data for future longitudinal studies to verify causal relationships with SO. Despite these limitations, we determined that total calorie intake and CHO intake per body weight could be useful parameters for managing SO.

## 5. Conclusions

Total calorie intake and CHO intake per body weight are inversely related to SO. CHO intake per body weight offers the highest predictability for SO among patterns of macronutrient intake, including total calorie intake. CHO intake per body weight could be an index worth investigating in the future for its association with SO. Since the influence of macronutrient intake patterns on SO varied between men and women, tailored nutritional interventions should be created based on sex. It is necessary to consider CHO intake per body weight as a compelling factor to prevent SO. Further research should be performed to identify whether patterns of macronutrient intake affect the development of SO. Prospective studies are also warranted to confirm the association between CHO intake per body weight and the improvement of SO in the future. Randomized controlled trials could also be considered to identify the effect of CHO intake per body weight based on nutritional interventions on SO. Experimental studies to verify the mechanisms through which CHO intake affects SO also should be performed.

## Figures and Tables

**Table 1:** Clinical characteristics of the older adults with or without sarcopenic obesity.

	Men			Women		
	Without SO	With SO	*p*	Without SO	With SO	*p* *
Unweighted number, *n*	1463	172		1858	335	
Age, years	72.0 ± 0.2	72.9 ± 0.5	0.083	73.1 ± 0.2	73.5 ± 0.4	0.432
BMI, kg/m^2^	22.6 ± 0.1	27.1 ± 0.1	<0.001	23.4 ± 0.1	27.8 ± 0.2	<0.001
Waist circumference, cm	83.6 ± 0.3	94.8 ± 0.5	<0.001	81.9 ± 0.3	91.7 ± 0.5	<0.001
Abdominal obesity, % (SE)	23.5 (1.4)	81.3 (3.7)	<0.001	36.7 (1.4)	81.7 (2.6)	<0.001
MBP, mmHg	94.4 ± 0.4	96.7 ± 0.9	0.020	95.1 ± 0.3	95.8 ± 0.8	0.445
Regular exercise, % (SE)	20.4 (1.6)	23.5 (4.1)	0.488	16.9 (1.2)	9.6 (1.7)	0.002
Smoking status, % (SE)			0.173			0.410
Never smoker	16.1 (1.2)	18.3 (3.2)		88.6 (1.0)	91.1 (1.8)	
Former smoker	28.6 (1.5)	33.6 (4.5)		2.3 (0.6)	2.1 (0.8)	
Someday smoker	30.2 (1.6)	31.9 (4.5)		3.2 (0.5)	3.6 (1.2)	
Every day smoker	25.1 (1.3)	16.2 (3.2)		5.9 (0.7)	3.3 (1.0)	
Alcohol intake, g/day	8.8 ± 0.4	7.7 ± 1.3	0.391	0.8 ± 0.1	0.5 ± 0.2	0.208
Glucose, mg/dL	104.0 ± 0.8	111.2 ± 2.4	0.004	104.4 ± 0.9	109.0 ± 1.5	0.010
Total cholesterol, mg/dL	180.4 ± 1.2	185.1 ± 3.4	0.183	198.2 ± 1.1	207.3 ± 2.8	0.003
Total calorie intake, kcal/day	1881.3 ± 26.1	1833.1 ± 52.8	0.429	1428.9 ± 14.8	1338.2 ± 32.0	0.007
Percent of CHO intake, %	71.5 ± 0.4	70.1 ± 1.0	0.201	77.7 ± 0.3	76.1 ± 0.6	0.014
Percent of protein intake, %	13.2 ± 0.1	13.5 ± 0.3	0.343	12.3 ± 0.1	12.8 ± 0.3	0.065
Percent of fat intake, %	12.2 ± 0.2	13.4 ± 0.6	0.068	10.4 ± 0.2	11.6 ± 0.4	0.015
CHO intake, g/kg/day	5.4 ± 0.1	4.4 ± 0.1	<0.001	5.2 ± 0.1	4.2 ± 0.1	<0.001
Protein intake, g/kg/day	1.0 ± 0.0	0.9 ± 0.0	0.001	0.8 ± 0.0	0.7 ± 0.0	<0.001
Fat intake, g/kg/day	0.4 ± 0.0	0.4 ± 0.0	0.384	0.3 ± 0.0	0.3 ± 0.0	0.213
Skeletal muscle mass index	0.861 ± 0.003	0.723 ± 0.004	<0.001	0.578 ± 0.002	0.464 ± 0.003	<0.001
Number of chronic diseases, % (SE)			<0.001			<0.001
0	63.1 (1.6)	41.4 (5.0)		65.2 (1.5)	52.2 (3.4)	
1	26.3 (1.4)	38.4 (5.0)		28.3 (1.3)	35.3 (3.3)	
≥2	10.6 (1.0)	20.2 (4.1)		6.5 (0.7)	12.5 (2.1)	

* Derived from weighted generalized linear regression analysis for continuous variables and weighted chi-square test for categorical variables. Abbreviations: SO, sarcopenic obesity; BMI, body mass index; MBP, mean blood pressure; CHO, carbohydrate; HTN, hypertension; DM, diabetes mellitus.

**Table 2:** Weighted logistic regression analysis showing relationship between macronutrient intake and sarcopenic obesity.

	Sarcopenic Obesity					
	Crude Model			Adjusted Model *		
	OR	95% CI	*p*	OR	95% CI	*p*
Men						
Total calorie intake (kcal/day) per 100 increment	0.99	0.96–1.02	0.438	0.99	0.95–1.04	0.761
Protein intake (%) per 1 increment	1.02	0.98–1.07	0.334	1.01	0.95–1.07	0.790
CHO intake (%) per 1 increment	0.99	0.98–1.00	0.183	0.99	0.97–1.01	0.234
Fat intake (%) per 1 increment	1.02	1.00–1.05	0.049	1.02	0.99–1.05	0.255
Protein intake per body weight (g/kg/day) per 1 increment	0.51	0.32–0.81	0.005	1.13	0.65–1.99	0.660
CHO intake per body weight (g/kg/day) per 1 increment	0.68	0.60–0.78	<0.001	0.93	0.80–1.09	0.378
Fat intake per body weight (g/kg/day) per 1 increment	0.79	0.45–1.39	0.416	1.56	0.80–3.03	0.190
Women						
Total calorie intake (kcal/day) per 100 increment	0.96	0.93–0.99	0.011	0.95	0.91–0.99	0.007
Protein intake (%) per 1 increment	1.04	1.00–1.08	0.049	1.04	0.99–1.08	0.098
CHO intake (%) per 1 increment	0.98	0.67–0.99	0.010	0.99	0.97–1.01	0.200
Fat intake (%) per 1 increment	1.03	1.01–1.05	0.009	1.01	0.99–1.04	0.277
Protein intake per body weight (g/kg/day) per 1 increment	0.43	0.67–0.70	0.001	0.78	0.47–1.31	0.344
CHO intake per body weight (g/kg/day) per 1 increment	0.70	0.64–0.77	<0.001	0.83	0.74–0.94	0.003
Fat intake per body weight (g/kg/day) per 1 increment	0.70	0.39–1.27	0.239	0.97	0.51–1.86	0.933

* Adjusted for age, waist circumference, regular exercise, smoking status, amount of alcohol intake, MBP, FPG, serum total cholesterol level, and number of chronic diseases. Abbreviations: OR, odds ratio; CI, confidence interval; CHO, carbohydrate; MBP, mean blood pressure; FPG, fasting plasma glucose.

## Data Availability

The data used in this study are available for free on the KNHANES website (http://knhanes.cdc.go.kr, accessed on 18 September 2021) for academic research purposes.

## Supplementary Materials

The following are available online at https://www.mdpi.com/article/10.3390/nu13114031/s1. Table S1: Weighted logistic regression analysis showing relationship between macronutrient intake and LSMI/obesity. Table S2: Correlation between carbohydrate intake per body weight and the other macronutrient intake pattern.

## References

[1] Population Ageing and Development 2012. https://www.un.org/en/development/desa/population/publications/ageing/population-ageing-development-2012.asp.

[2] Population Projections for Korea (2017–2067). http://kostat.go.kr/portal/eng/pressReleases/8/8/index.board?bmode=download&bSeq=&aSeq=375684&ord=2.

[3] Raguso C.A., Kyle U., Kossovsky M.P., Roynette C., Paoloni-Giacobino A., Hans D., Genton L., Pichard C. (2006). A 3-year longitudinal study on body composition changes in the elderly: Role of physical exercise. Clin. Nutr..

[4] Santanasto A.J., Goodpaster B.H., Kritchevsky S.B., Miljkovic I., Satterfield S., Schwartz A.V., Cummings S.R., Boudreau R.M., Harris T.B., Newman A.B. (2017). Body composition remodeling and mortality: The health aging and body composition study. J. Gerontol. A Biol. Sci. Med. Sci..

[5] Ponti F., Santoro A., Mercatelli D., Gasperini C., Conte M., Martucci M., Sangiorgi L., Franceschi C., Bazzocchi A. (2019). Aging and imaging assessment of body composition: From fat to facts. Front. Endocrinol. (Lausanne).

[6] Choi K.M. (2016). Sarcopenia and sarcopenic obesity. Korean J. Intern. Med..

[7] Huang P., Luo K., Xu J., Huang W., Yin W., Xiao M., Wang Y., Ding M., Huang X. (2021). Sarcopenia as a risk factor for future hip fracture: A Meta-analysis of prospective cohort studies. J. Nutr. Health Aging.

[8] Chen H., Ma J., Liu A., Cui Y., Ma X. (2020). The association between sarcopenia and fracture in middle-aged and elderly people: A systematic review and meta-analysis of cohort studies. Injury.

[9] Dwivedi A.K., Dubey P., Cistola D.P., Reddy S.Y. (2020). Association between obesity and cardiovascular outcomes: Updated evidence from meta-analysis studies. Curr. Cardiol. Rep..

[10] Lee J.J., Beretvas S.N., Freeland-Graves J.H. (2014). Abdominal adiposity distribution in diabetic/prediabetic and nondiabetic populations: A meta-analysis. J. Obes..

[11] Zhou W., Shi Y., Li Y.Q., Ping Z., Wang C., Liu X., Lu J., Mao Z.X., Zhao J., Yin L. (2018). Body mass index, abdominal fatness, and hypertension incidence: A dose-response meta-analysis of prospective studies. J. Hum. Hypertens..

[12] Zamboni M., Mazzali G., Fantin F., Rossi A., Di Francesco V. (2008). Sarcopenic obesity: A new category of obesity in the elderly. Nutr. Metab. Cardiovasc. Dis..

[13] Wang H., Hai S., Liu Y.X., Cao L., Liu Y., Liu P., Yang Y., Dong B.R. (2019). Associations between sarcopenic obesity and cognitive impairment in elderly chinese community-dwelling individuals. J. Nutr. Health Aging.

[14] Kokkeler K.J.E., van den Berg K.S., Comijs H.C., Oude Voshaar R.C., Marijnissen R.M. (2019). Sarcopenic obesity predicts nonremission of late-life depression. Int. J. Geriatr. Psychiatry.

[15] Atkinson R.L., Butterfield G., Dietz W., Fernstrom J., Frank A., Hansen B., Moore B. (2004). Weight management: State of the science and opportunities for military programs. Institute of Medicine (US) Subcommittee on Military Weight Management.

[16] Paddon-Jones D., Rasmussen B.B. (2009). Dietary protein recommendations and the prevention of sarcopenia. Curr. Opin. Clin. Nutr. Metab. Care.

[17] Jane L., Atkinson G., Jaime V., Hamilton S., Waller G., Harrison S. (2015). Intermittent fasting interventions for the treatment of overweight and obesity in adults aged 18 years and over: A systematic review protocol. JBI Database Syst. Rev. Implement. Rep..

[18] Barnosky A.R., Hoddy K.K., Unterman T.G., Varady K.A. (2014). Intermittent fasting vs daily calorie restriction for type 2 diabetes prevention: A review of human findings. Transl. Res..

[19] Xiao Q., Garaulet M., Scheer F. (2019). Meal timing and obesity: Interactions with macronutrient intake and chronotype. Int. J. Obes..

[20] Anderson J.W., Baird P., Davis R.H., Ferreri S., Knudtson M., Koraym A., Waters V., Williams C.L. (2009). Health benefits of dietary fiber. Nutr. Rev..

[21] Sartorius K., Sartorius B., Madiba T.E., Stefan C. (2018). Does high-carbohydrate intake lead to increased risk of obesity? A systematic review and meta-analysis. BMJ Open.

[22] Jung H.W., Kim S.W., Kim I.Y., Lim J.Y., Park H.S., Song W., Yoo H.J., Jang H.C., Kim K., Park Y. (2018). Protein intake recommendation for korean older adults to prevent sarcopenia: Expert consensus by the Korean Geriatric Society and the Korean Nutrition Society. Ann. Geriatr. Med. Res..

[23] Porter Starr K.N., Pieper C.F., Orenduff M.C., McDonald S.R., McClure L.B., Zhou R., Payne M.E., Bales C.W. (2016). Improved function with enhanced protein intake per meal: A pilot study of weight reduction in frail, obese older adults. J. Gerontol. A Biol. Sci. Med. Sci..

[24] Petroni M.L., Caletti M.T., Dalle Grave R., Bazzocchi A., Aparisi Gómez M.P., Marchesini G. (2019). Prevention and treatment of sarcopenic obesity in women. Nutrients.

[25] Park Y., Choi J.E., Hwang H.S. (2018). Protein supplementation improves muscle mass and physical performance in undernourished prefrail and frail elderly subjects: A randomized, double-blind, placebo-controlled trial. Am. J. Clin. Nutr..

[26] Kim B.Y., Kang S.M., Kang J.H., Kang S.Y., Kim K.K., Kim K.B., Kim B., Kim S.J., Kim Y.H., Kim J.H. (2021). 2020 Korean society for the study of obesity guidelines for the management of obesity in Korea. J. Obes. Metab. Syndr..

[27] Kweon S., Kim Y., Jang M.-j., Kim Y., Kim K., Choi S., Chun C., Khang Y.-H., Oh K. (2014). Data resource profile: The Korea National Health and Nutrition Examination Survey (KNHANES). Int. J. Epidemiol..

[28] Kim Y. (2014). The Korea National Health and Nutrition Examination Survey (KNHANES): Current status and challenges. Epidemiol. Health.

[29] Deierlein A.L., Morland K.B., Scanlin K., Wong S., Spark A. (2014). Diet quality of urban older adults age 60 to 99 years: The cardiovascular health of seniors and built environment study. J. Acad. Nutr. Diet..

[30] Madden J.P., Goodman S.J., Guthrie H.A. (1976). Validity of the 24-hr. recall. Analysis of data obtained from elderly subjects. J. Am. Diet. Assoc..

[31] Kwon Y.-J., Lee H.S., Park J.-Y., Lee J.-W. (2020). Associating intake proportion of carbohydrate, fat, and protein with all-cause mortality in Korean adults. Nutrients.

[32] Seo M.H., Lee W.-Y., Kim S.S., Kang J.-H., Kang J.-H., Kim K.K., Kim B.-Y., Kim Y.-H., Kim W.-J., Kim E.M. (2019). 2018 Korean society for the study of obesity guideline for the management of obesity in Korea. J. Obes. Metab. Syndr..

[33] Studenski S.A., Peters K.W., Alley D.E., Cawthon P.M., McLean R.R., Harris T.B., Ferrucci L., Guralnik J.M., Fragala M.S., Kenny A.M. (2014). The FNIH sarcopenia project: Rationale, study description, conference recommendations, and final estimates. J. Gerontol. Ser. A Biol. Sci. Med. Sci..

[34] Lee S.Y., Park H.S., Kim D.J., Han J.H., Kim S.M., Cho G.J., Kim D.Y., Kwon H.S., Kim S.R., Lee C.B. (2007). Appropriate waist circumference cutoff points for central obesity in Korean adults. Diabetes Res. Clin. Pract..

[35] Craig C.L., Marshall A.L., Sjöström M., Bauman A.E., Booth M.L., Ainsworth B.E., Pratt M., Ekelund U., Yngve A., Sallis J.F. (2003). International physical activity questionnaire: 12-country reliability and validity. Med. Sci. Sports Exerc..

[36] Charlson M.E., Pompei P., Ales K.L., MacKenzie C.R. (1987). A new method of classifying prognostic comorbidity in longitudinal studies: Development and validation. J. Chronic Dis..

[37] Manore M.M. (2005). Exercise and the Institute of Medicine recommendations for nutrition. Curr. Sports Med. Rep..

[38] Haizlip K.M., Harrison B.C., Leinwand L.A. (2015). Sex-based differences in skeletal muscle kinetics and fiber-type composition. Physiology.

[39] Horton T.J., Dow S., Armstrong M., Donahoo W.T. (2009). Greater systemic lipolysis in women compared with men during moderate-dose infusion of epinephrine and/or norepinephrine. J. Appl. Physiol..

[40] Karastergiou K., Smith S.R., Greenberg A.S., Fried S.K. (2012). Sex differences in human adipose tissues—The biology of pear shape. Biol. Sex Differ..

[41] Okamura T., Miki A., Hashimoto Y., Kaji A., Sakai R., Osaka T., Hamaguchi M., Yamazaki M., Fukui M. (2019). Shortage of energy intake rather than protein intake is associated with sarcopenia in elderly patients with type 2 diabetes: A cross-sectional study of the KAMOGAWA-DM cohort. J Diabetes.

[42] Kye S., Kwon S.O., Lee S.Y., Lee J., Kim B.H., Suh H.J., Moon H.K. (2014). Under-reporting of energy intake from 24-hour dietary recalls in the Korean National Health and Nutrition Examination Survey. Osong Public Health Res. Perspect..

[43] Kim S., Park H., Yun H., Lee B., Park C.Y. (2020). Accuracy of 24-hour diet recalls in older Korean women. Curr. Dev. Nutr..

[44] Zafar M.I., Mills K.E., Zheng J., Peng M.M., Ye X., Chen L.L. (2019). Low glycaemic index diets as an intervention for obesity: A systematic review and meta-analysis. Obes. Rev..

[45] Schwingshackl L., Hoffmann G. (2013). Long-term effects of low glycemic index/load vs. high glycemic index/load diets on parameters of obesity and obesity-associated risks: A systematic review and meta-analysis. Nutr. Metab. Cardiovasc. Dis..

[46] Lim M.T., Pan B.J., Toh D.W.K., Sutanto C.N., Kim J.E. (2021). Animal protein versus plant protein in supporting lean mass and muscle strength: A systematic review and meta-analysis of randomized controlled trials. Nutrients.

