# Supplementary material for: Using Dietary Macronutrient Patterns to Predict Sarcopenic Obesity in Older Adults: A Representative Korean Nationwide Population-Based Study
Source: Nutrients. 2021 Nov 11;13(11):4031. doi: 10.3390/nu13114031 (PMC8625406; doi:10.3390/nu13114031)
Supplement: Supplementary file 1 [file nutrients-13-04031-s001.zip › nutrients-1448364-supplementary.pdf]

**Table S1.** Weighted logistic regression analysis showing relationship between macronutrient intake and LSMI/obesity

| | LSMI | | | | | | Obesity | | | | | |
|---|---|---|---|---|---|---|---|---|---|---|---|---|
| | Crude model | | | Adjusted model* | | | Crude model | | | Adjusted model* | | |
| | OR | 95% CI | *p* | OR | 95% CI | *p* | OR | 95% CI | *p* | OR | 95% CI | *p* |
| Men | | | | | | | | | | | | |
| Total calorie intake (kcal/day) | 0.95 | 0.93–0.97 | < 0.001 | 0.97 | 0.94–0.99 | 0.018 | 1.02 | 1.00–1.04 | 0.044 | 1.01 | 0.97–1.05 | 0.585 |
| CHO intake per body weight (g/kg/day) per 1 increment | 0.85 | 0.78–0.93 | 0.001 | 0.98 | 0.88–1.08 | 0.650 | 0.71 | 0.62–0.82 | < 0.001 | 0.84 | 0.73–0.97 | 0.018 |
| Women | | | | | | | | | | | | |
| Total calorie intake (kcal/day) | 0.95 | 0.93–0.98 | < 0.001 | 0.95 | 0.93–0.98 | 0.001 | 1.02 | 1.00–1.04 | 0.111 | 0.98 | 0.94–1.01 | 0.147 |
| CHO intake per body weight (g/kg/day) per 1 increment | 0.86 | 0.80–0.92 | < 0.001 | 0.95 | 0.88–1.04 | 0.254 | 0.72 | 0.67–0.77 | < 0.001 | 0.80 | 0.72–0.89 | < 0.001 |

*Adjusted for age, waist circumference, regular exercise, smoking status, amount of alcohol intake, MBP, FPG, serum total cholesterol level, and number of chronic diseases.

Abbreviations: LSMI, low skeletal muscle mass index; OR, odds ratio; CI, confidence interval; CHO, carbohydrate; MBP, mean blood pressure; FPG, fasting plasma glucose

**Table S2.** Correlation between carbohydrate intake per body weight and the other macronutrient intake pattern

| | CHO intake per body weight (g/kg/day) | |
|---|---|---|
| | $r^*$ | $p$ |
| Men | | |
| Total calorie intake (kcal/day) | 0.685 | < 0.001 |
| Protein intake (%) | -0.092 | < 0.001 |
| CHO intake (%) | 0.225 | < 0.001 |
| Fat intake (%) | -0.146 | < 0.001 |
| Protein intake per body weight (g/kg/day) | 0.556 | < 0.001 |
| Fat intake per body weight (g/kg/day) | 0.249 | < 0.001 |
| Women | | |
| Total calorie intake (kcal/day) | 0.811 | < 0.001 |
| Protein intake (%) | -0.140 | < 0.001 |
| CHO intake (%) | 0.219 | < 0.001 |
| Fat intake (%) | -0.194 | < 0.001 |
| Protein intake per body weight (g/kg/day) | 0.576 | < 0.001 |
| Fat intake per body weight (g/kg/day) | 0.263 | < 0.001 |

*Correlation coefficient between CHO intake per body weight and each macronutrient intake pattern using Pearson's correlation test.

Abbreviation: CHO, carbohydrate.
